# Identifying the nature of surface chemical modification for directed self-assembly of block copolymers

**DOI:** 10.3762/bjnano.8.198

**Published:** 2017-09-21

**Authors:** Laura Evangelio, Federico Gramazio, Matteo Lorenzoni, Michaela Gorgoi, Francisco Miguel Espinosa, Ricardo García, Francesc Pérez-Murano, Jordi Fraxedas

**Affiliations:** 1 Institut de Microelectrònica de Barcelona (IMB-CNM, CSIC), C/Til·lers, Campus Universitat Autònoma de Barcelona, Cerdanyola del Vallès, Barcelona, Spain; 2 Catalan Institute of Nanoscience and Nanotechnology (ICN2), CSIC and BIST, Campus UAB, Bellaterra, 08193, Barcelona, Spain; 3 Institute for Nanospectroscopy, Energy Materials In-situ Laboratory (EMIL), Helmholtz Zentrum Berlin für Materialien und Energie GmbH, Albert Einsteinstrasse 15, 12489 Berlin, Germany; 4 Instituto de Ciencia de Materiales de Madrid (ICMM-CSIC), Sor Juana Inés de la Cruz, 3, Cantoblanco, 28049 Madrid, Spain

**Keywords:** block copolymer, chemical guiding pattern, directed self-assembly, thin film, X-ray photoemission spectroscopy

## Abstract

In recent years, block copolymer lithography has emerged as a viable alternative technology for advanced lithography. In chemical-epitaxy-directed self-assembly, the interfacial energy between the substrate and each block copolymer domain plays a key role on the final ordering. Here, we focus on the experimental characterization of the chemical interactions that occur at the interface built between different chemical guiding patterns and the domains of the block copolymers. We have chosen hard X-ray high kinetic energy photoelectron spectroscopy as an exploration technique because it provides information on the electronic structure of buried interfaces. The outcome of the characterization sheds light onto key aspects of directed self-assembly: grafted brush layer, chemical pattern creation and brush/block co-polymer interface.

## Introduction

Directed self-assembly (DSA) of block copolymers (BCPs) is a chemical-based complementary alternative to traditional patterning methods providing sub-10 nm resolution, low-cost processing and high throughput [1–3]. Moreover, it is one of the most promising techniques for the development of the next generation of nanoelectronic devices and circuits, as it is compatible with current manufacturing processes.

BCPs are macromolecules derived from more than one species of monomers with inter-monomer covalent bonding. Due to the repulsion between different blocks, the BCPs tend to segregate and undergo a separation phase with controllable dimensions and functionalities due to unfavorable enthalpic interactions [4]. The global parameters that govern the phase behavior of BCPs are given by the χ·*N* product, where χ stands for the Flory Huggins parameter and *N* the number of statistical segments in a BCP chain, which is related to the free energy of the system and the composition of the blocks [5–6]. When the BCP self-assembly is used in combination with surface prepatterning, aligned structures of alternative phases of the blocks can be obtained. This is the principle of DSA. The main advantages are a relaxation of the resolution requirements of traditional lithography methods, as the period of the prepattern can be larger than the final period of the self-assembled pattern, and an improvement in both line-edge and line-width roughness.

There are mainly two techniques to direct the self-assembly of BCPs: graphoepitaxy and chemical epitaxy. In graphoepitaxy, the BCPs are aligned by a topographical substrate pattern[7–9]**,** whereas in chemical epitaxy the self-assembly is driven by the difference of surface free-energies between the domains of the copolymer and the chemical prepattern[2,10–12]. Currently, the industry is more focused on chemical epitaxy rather than on graphoepitaxy due to the fact that the BCP is guided in with negligible changes in the height step of the patterns and because of its easier integration[13].

In chemical epitaxy DSA, the interfacial energies between each domain of the copolymer and the chemically patterned surfaces strongly influence the final morphology and micro-domain ordering. Therefore, an accurate control of the surface chemistry is needed, for example, to obtain the desirable orientation during self-assembly (parallel or perpendicular lamellae or cylinders), to avoid dewetting phenomena or to minimize the presence of defects. Generally, in chemical epitaxy DSA, the background (unmodified) surface should be slightly attractive to one of the domains of the copolymers while the chemically modified areas should be slightly attractive to the other one.

In order to understand the resulting BCP morphology when it is self-assembled on the top of a chemical guiding pattern, it is important to determine which chemical interactions occur between both modified and unmodified regions of the substrate with each block of the copolymer. One technique especially suited for the characterization of buried interfaces is hard X-ray high kinetic energy photoelectron spectroscopy (HAXPES) [14]. Photoemission is a well-known technique which provides information on the electronic structure of surfaces. Its high surface sensitivity arises from the small mean free path of the outcoming photoelectrons in solid matter. Using conventional excitation sources, kinetic energies below 1500 eV can be achieved, which correspond approximately to a 2 nm probing depth for inorganic materials. The possibility of acquiring photoemission spectra at higher kinetic energies, as high as 10 keV, has permitted the exploration of the chemical environment of subsurface regions down to more than 20 nm for polymeric materials [15]. HAXPES reaches its full potential when using synchrotron radiation as an excitation source since, in this case, photon energy (and thus kinetic energy) can be tuned so that the probing depth can be also varied in a controlled and continuous manner. Nevertheless, one drawback of this technique, in particular when using polymers and organic materials, is the irreversible damage caused by the impinging beam. Therefore, this is a matter that has to be carefully addressed in any measurement, even for inorganic materials[16–17].

**Figure 1:**
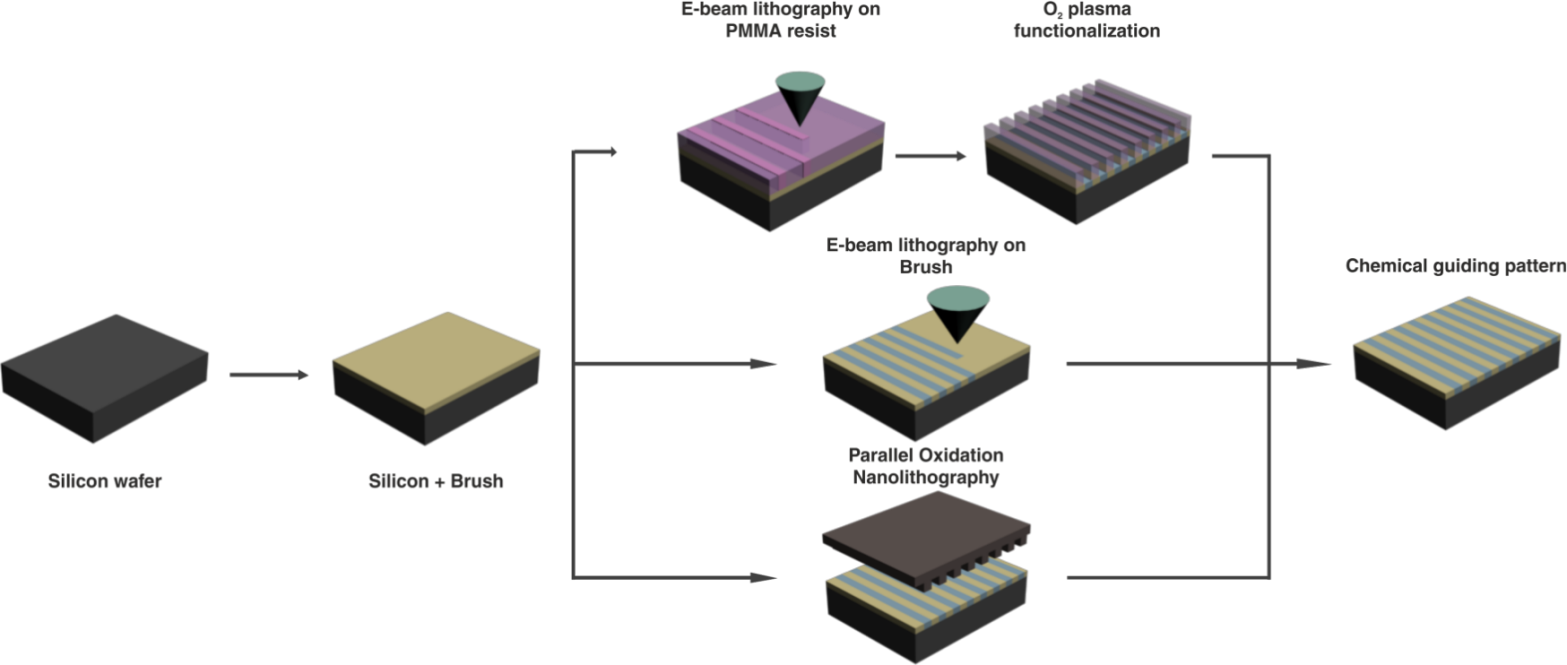
Schematized description of the selected chemical epitaxy DSA processes performed on grafted brush layer on a silicon substrate. The method in the top part of the figure uses EBL on a PMMA resist and ulterior oxygen plasma functionalization (two-step process), while the other two nanolithography methods (middle and bottom) are performed in absence of a resist (direct writing).

Here, we have used HAXPES as well as conventional XPS to investigate the chemical changes that occur during the processing steps involved in DSA. As it is depicted in Figure 1, three chemical epitaxy DSA processes have been investigated in order to determine the dominant interactions between the substrate (a brush layer covering a silicon wafer, left in the Figure) and the block copolymer domains. The first DSA (two steps) process uses electron beam lithography (EBL) [12] on a poly(methyl methacrylate) (PMMA) resist with a subsequent substrate functionalization with oxygen plasma of the uncovered areas (top of Figure 1). The two other processes are based on direct writing (one-step) methods, thus avoiding the use of a resist. The selected methods are EBL (middle in Figure 1) and parallel oxidation nanolithography [18] (PON) (bottom of the Figure), respectively. The PON method is performed by contacting a conductive mold with the brush surface while applying a voltage under high humidity conditions. Details on the preparation methods can be found in the Experimental section.

## Results and Discussion

### Two-step electron beam and oxygen plasma modification

**Figure 2:**
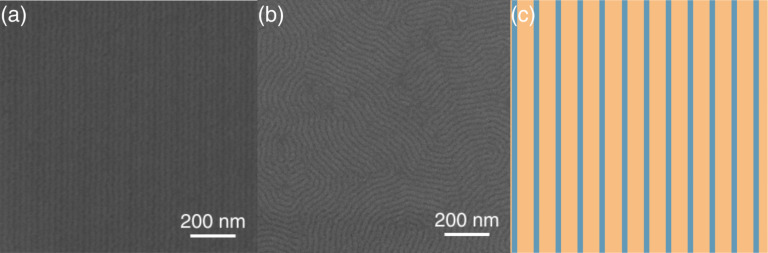
SEM images of PS-*b*-PMMA BCP after removal of the PMMA blocks prepared after EBL and oxygen plasma functionalization of grafted PS–OH layers deposited on silicon wafers (see Figure 1) after (a) annealing at 230 °C and cooling in nitrogen and (b) annealing at 260 °C and cooling in air. (c) Scheme of the induced patterns (same scale as in (a) and (b)).

Figure 2 shows SEM images of directed self-assembled films of PS-*b*-PMMA BCP prepared on substrates modified with EBL and oxygen plasma and annealed using the two selected annealing processes described above: (Figure 2a) 230 °C in nitrogen atmosphere for 5 min and cooling in nitrogen and (Figure 2b) 260 °C in nitrogen atmosphere for 5 min and cooling in air. A scheme with the same scale of the distribution of the generated patterns is shown in the right of the figure as a guide. Before imaging, the PMMA blocks were removed by exposing the sample to 50 sccm of oxygen flow at 500 W for 18 s in order to visualize the efficiency of the DSA process. From the figure, it becomes evident that when the brush cooling is performed in nitrogen rather than in air, the polymer has the proper surface free energy to induce the alignment of the BCP after the lithography and BCP spin-coating.

**Figure 3:**
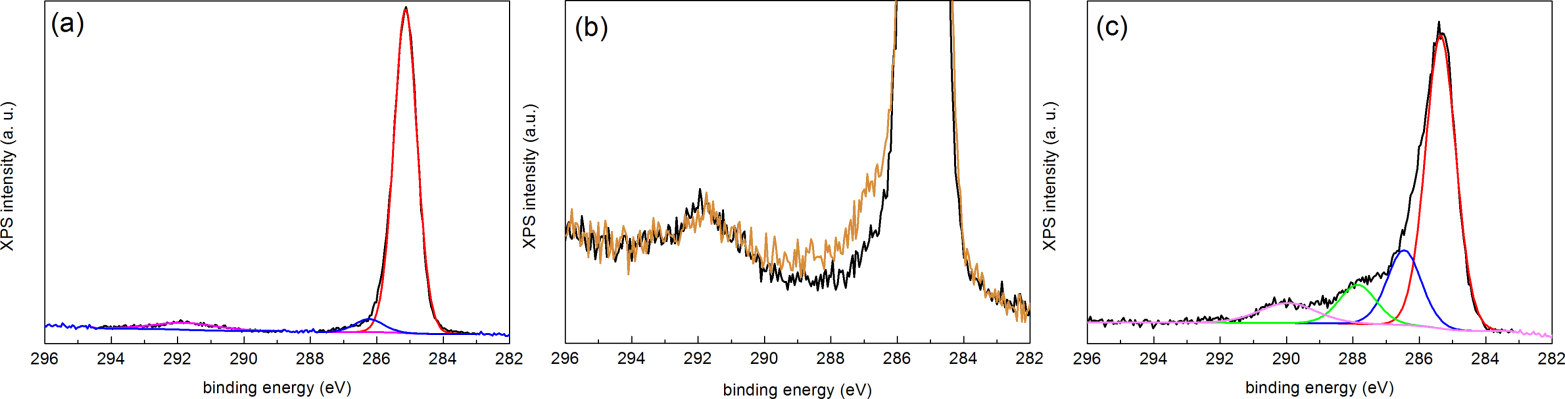
HAXPES spectra corresponding to the C 1s region of grafted PS–OH samples (a) cooled in nitrogen, (b) cooled in air (orange) and in nitrogen (black) and (c) cooled in nitrogen and exposed to oxygen plasma, taken with 2020 eV using a SCIENTA R4000 hemispherical analyser. Least-square fits of the experimental data (continuous black line) after background subtraction (Shirley-type) are shown in (a) and (c) using a combination of Gaussian (70%) and Lorentzian (30%) functions under the constraint of identical full width at half maximum (FWHM) values for all components.

In order to understand the origin of the influence of the DSA process, we have performed HAXPES experiments on PS–OH grafted layers cooled under the selected conditions. Figure 3a shows the experimental HAXPES C 1s line (continuous black line) of the sample cooled in nitrogen together with a least-square fit after background subtraction (see caption of figure for details). The most prominent line (continuous red line), with a binding energy of 285.1 eV corresponds to C–C and C–H bonding. The continuous blue line in Figure 3a, with a binding energy of 286.2 eV, corresponds to the hydroxyl bonding of the PS–OH. The π–π* shake-up feature at 291.9 eV, characteristic of a pure PS spectrum (continuous magenta line) [19], is also observed. Figure 3b compares the zoomed in spectra of the C 1s lines corresponding to the sample cooled in air (orange) and that cooled in nitrogen (black), respectively. The figure evidences a small but clear increase in intensity of the region corresponding to hydroxyl bonding for the sample cooled in air. We point out that this minor effect in the C 1s line can only be observed because of the high-energy resolution used in the HAXPES experiments and that parallel XPS measurements of samples prepared under the same conditions did not show any significant difference. The higher density of hydroxyl bonding induces higher attraction to PMMA blocks due to the affinity with carbonyl PMMA groups. In this case, the chemical guiding patterns created afterwards on the sample cooled in air will not be effective since the brush is already slightly PMMA affine before the oxygen plasma functionalization. Conversely, when the sample is cooled down in nitrogen, PS does not undergo oxidation. As a consequence, such a sample is slightly affine to PS before functionalization. When chemical guiding stripes are defined on this substrate by oxygen plasma exposure (see Figure 2), there will be enough chemical contrast to guide the alignment of the BCP. We thus conclude that such a small increase in hydroxyl bonding is sufficient to disable the alignment capabilities of the PS–OH brush layer (see Figure 2a,b).

Once the favourable cooling conditions for the preparation of the polymer brush layer were analysed, we investigated the effect of the functionalization upon exposure to oxygen plasma. Figure 3c shows the C 1s HAXPES spectrum taken with 2020 eV photons of the sample cooled in nitrogen after the oxygen plasma treatment. The comparison with Figure 3a evidences an increase in intensity towards higher binding energies in the ≈286–291 eV region, which corresponds to contributions from different carbon–oxygen bonding configurations, as a result of the effect of the oxygen plasma exposure on the PS–OH brush layer. The continuous red and blue lines, with binding energies of 285.3 and 286.5eV, respectively, exhibit a 0.2–0.3 eV shift towards higher energies as compared to Figure 3a, indicating different charging. The continuous blue line, corresponding to hydroxyl bonding, becomes more intense as compared to Figure 3a. Two new features are observed at 287.9 and 290 eV binding energies, which are assigned to the carbonyl (C–O, continuous green line) and carboxyl (O–C=O, continuous pink line) contributions, respectively. Thus, oxygen plasma activates the brush layer surface by creating a distribution of C–O bonding, while annealing and cooling in air induces essentially hydroxylation of the surface. Thus, the combination between optimal process conditions for grafting the polymer brush layer and an adequate chemical functionalization by oxygen plasma exposure leads to the possibility to generate efficient chemical patterns for guiding the self-assembly of the BCP.

**Figure 4:**
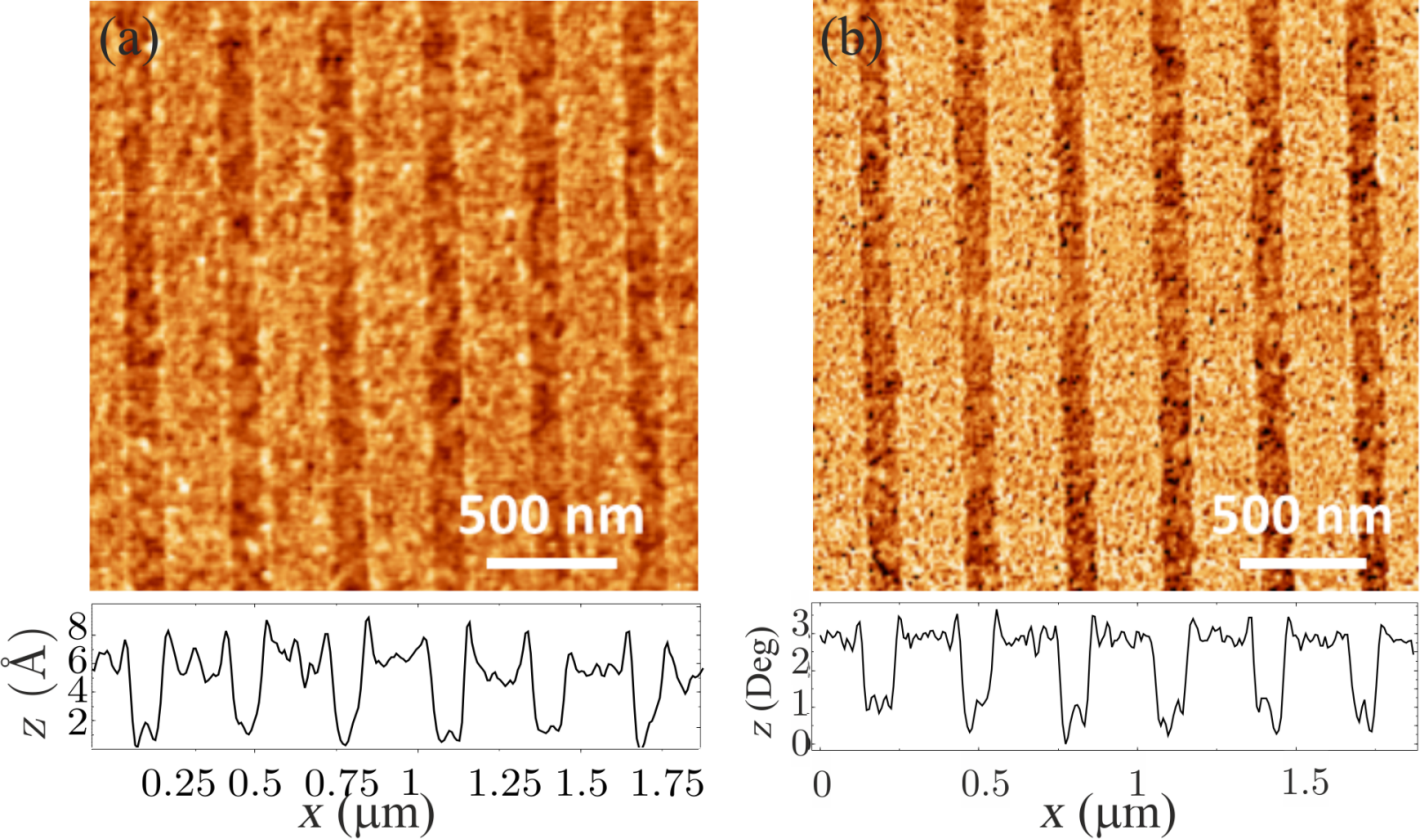
AFM topography (a) and phase (b) images of a chemical guiding pattern created by EBL followed by oxygen plasma modification on the brush sample cooled in nitrogen (see Figure 1). Representative cross sections are shown below both images.

Additional information can be obtained from AFM experiments. Figure 4a,b shows the AFM topography and phase images, respectively, of the chemical guiding patterns [20]. In both images the brighter lines correspond to the unexposed PS–OH brush layer, while the darker lines stand for the modified surface. The chemical contrast revealed by the AFM phase image is a signature of the fact that the unexposed PS–OH stripes are slightly affine to PS while the stripes exposed to oxygen plasma are slightly affine to PMMA due to the oxidation of the polymer. Applying the same method to create the guiding patterns on the sample cooled in air resulted in a surface which did not show any contrast when performing the AFM phase characterization. Furthermore, the oxygen plasma exposure induces a removal of about 0.4 nm of PS–OH, as deduced from the topography image in Figure 4a. This is in line with complementary X-ray reflectometry (XRR) results performed on annealed brush layers which deliver a brush thickness for unexposed and exposed surfaces of 4.6 nm and 3.9 nm, respectively. The presence of some topography between the stripes may enhance the guiding efficiency of the chemical patterns [7–9], but such generated corrugation is not enough to induce the alignment of the BCP. This is confirmed by the results shown in Figure 2a,b.

**Figure 5:**
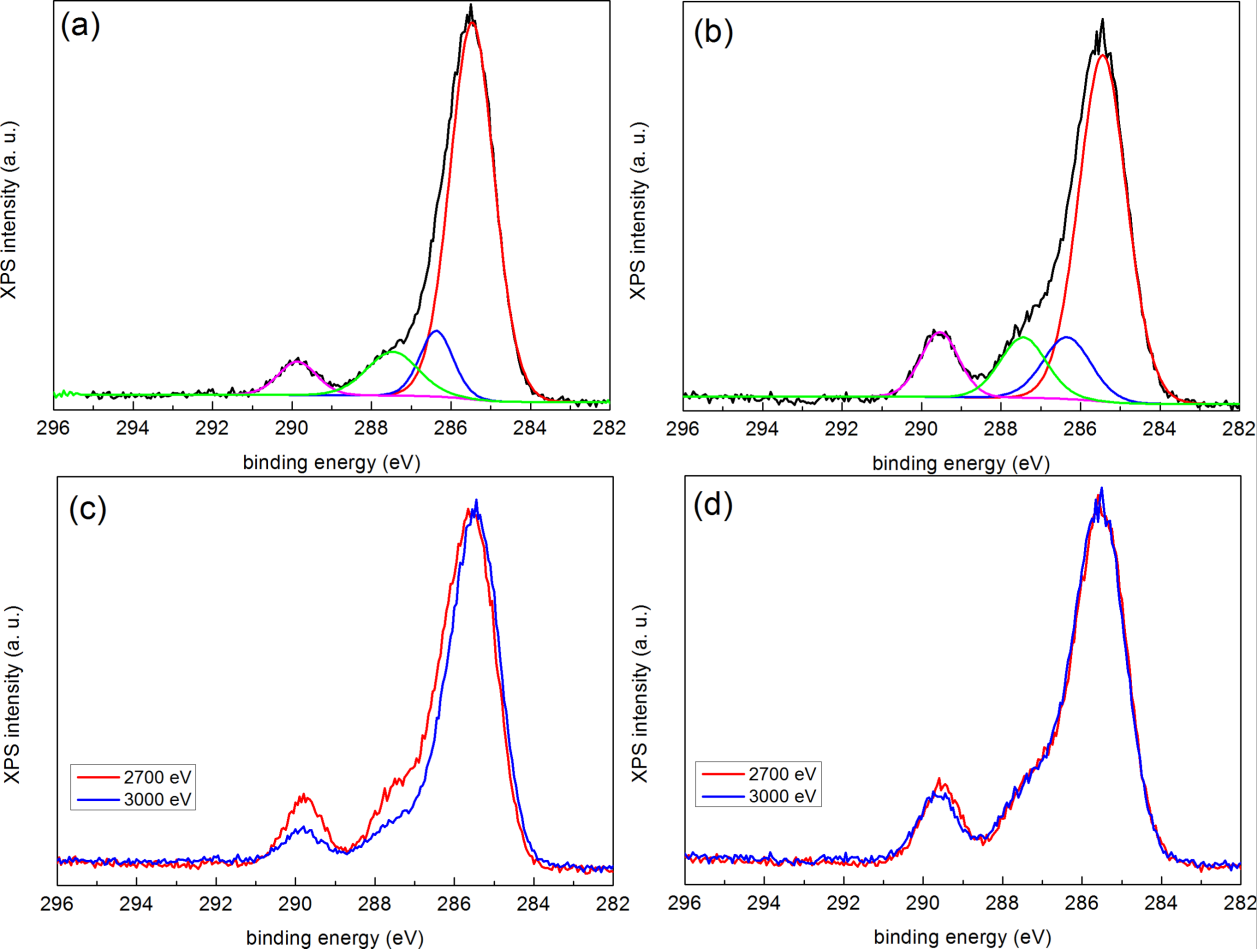
HAXPES spectra corresponding to the C 1s region of (a) unmodified and (b) modified samples, respectively, taken with 3000 eV photons including a deconvolution of the components using a least-square fit after background subtraction. Comparison of spectra taken with 2700 and 3000 eV photons of the unmodified (c) and modified (d) samples, respectively. Spectra in (c) and (d) have been normalized and aligned to the peak maxima.

Finally, we investigate the interfacial affinity between the PMMA block and both modified (oxygen plasma) and unmodified brush layers. For this matter ≈20 nm thick PMMA films have been deposited on top of grafted brush layers that have been unexposed and exposed to oxygen plasma, respectively, without prior EBL modification. Figure 5a,b shows the C 1s HAXPES spectra acquired with 3000 eV photons together with the deconvolution using least-square fits after background subtraction. Both spectra show the characteristic 287.5 eV (continuous green line) and 290 eV (continuous magenta line) peaks of PMMA, corresponding to O–CH_3_ and O–C=O configurations, respectively, with a 1:1 stoichiometric relationship [21]. The continuous red and blue lines correspond to C–C/C–H bonding and to hydroxyl bonding, respectively, as described in Figure 3. The nominal O–C=O contribution to the full photoemission spectrum in pure PMMA is 20% (one carbon over the total five carbons in the monomer). Such proportion measured with XPS and HAXPES from PMMA films directly deposited on silicon substrates (no brush layers) is about 17–18%, as obtained by comparing the area of the O–C=O contribution to the total area of the C 1s line. The lower proportion can be ascribed to contamination during exposure to air. From Figure 5a,b we observe that the proportions are about 7% and 13% for the unexposed and exposed samples, respectively. Apart from surface contamination, the lower values are due to the contribution from the underlying PS–OH brush layer, which adds to the main C–C/C–H line, and to a lesser extent, to the C–OH line. The lower the values, the larger the contribution from the brush layer, so that we can conclude that the unmodified brush layer is not uniformly covered by the relatively thick PMMA film as a result of the lower affinity between both materials (inefficient wetting). However, in the case of the sample exposed to oxygen plasma, the modified character of the brush layer triggers a higher affinity to PMMA (more efficient although non complete wetting).

This is further confirmed when the C 1s HAXPES spectra are taken at different photon energies, as shown in Figure 5c,d, where the spectra have been acquired at 2700 and 3000 eV, respectively. Increasing photon energy implies increasing kinetic energy and thus increasing probing depth. In the case of the unexposed sample, the mentioned proportion varies from 12% at 2700 eV to 7% at 3000 eV (5% decrease) and the modified brush layer from 15% at 2700 eV to 13% at 3000 eV (2% decrease). The calculated variations of the relative contribution of the O–C=O configuration between 2700 and 3000 eV is less than 1% using a two-layer model for a 20 nm PMMA film homogeneously covering a 5 nm thick brush layer (see Supporting Information File 1, Figure S1). Thus, the larger decrease can be ascribed to the increasing contribution of the incompletely covered PS–OH substrate.

### Direct writing

**Figure 6:**
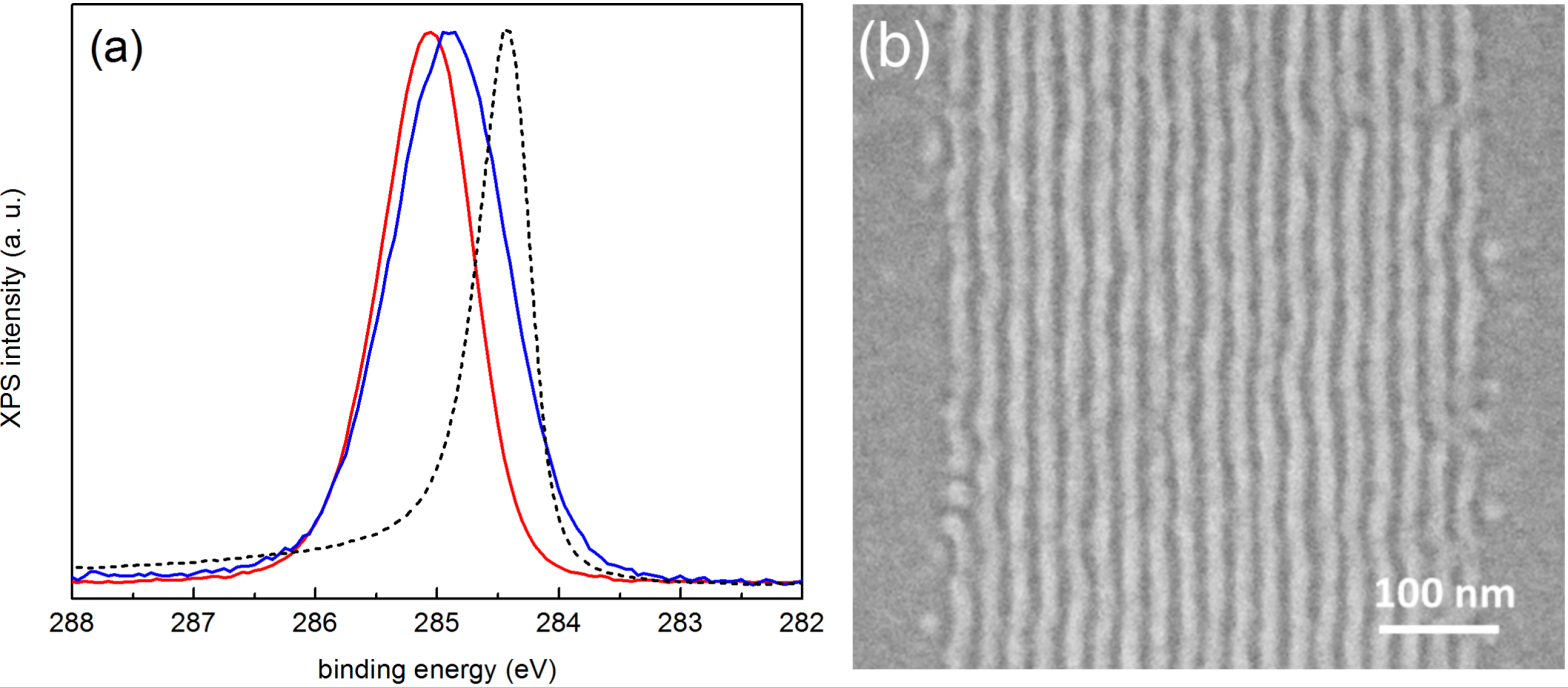
(a) C 1s XPS spectra of a grafted brush PS–OH layer after annealing at 230 °C and cooling in nitrogen (continuous red line), a sample modified by EBL (continuous blue line), and HOPG (discontinuous black line) using a PHOIBOS150 analyzer and monochromatic 1486.6 eV excitation. (b) SEM image of a 22 nm pitch PS-*b*-PMMA aligned in a pattern created by direct electron beam exposure.

We discuss here the results obtained with resistless lithography methods, namely EBL [12] and PON [18]. Figure 6a shows a comparison between C 1s XPS spectra of three PS–OH brush layer treated surfaces after annealing at 230 °C and cooling in nitrogen (continuous red line), after EBL (continuous blue line) and with a freshly cleaved highly-oriented pyrolytic graphite (HOPG) surface (discontinuous black line). The surface modified by EBL shows a relatively large broadening and a strong shift towards lower binding energies, as compared to the sample modified by EBL and oxygen plasma. Binding energies have been referenced to the Si 2p_3/2_ peak (99.3 eV) from the buried silicon substrate. The mentioned shift towards lower binding energies denotes the increasing presence of sp^2^ bonding based on the comparison with the results from a freshly cleaved HOPG sample (discontinuous black line), which shows a narrow peak centered at 284.4 eV, characteristic of sp^2^ bonding. Such increase in sp^2^ bonding is in line, although not a direct proof, with the cross-linking of PS due to electron beam exposure, as has been reported in the literature [22–23]. This contributes to the alignment of the BCP, as shown in Figure 6b.

**Figure 7:**
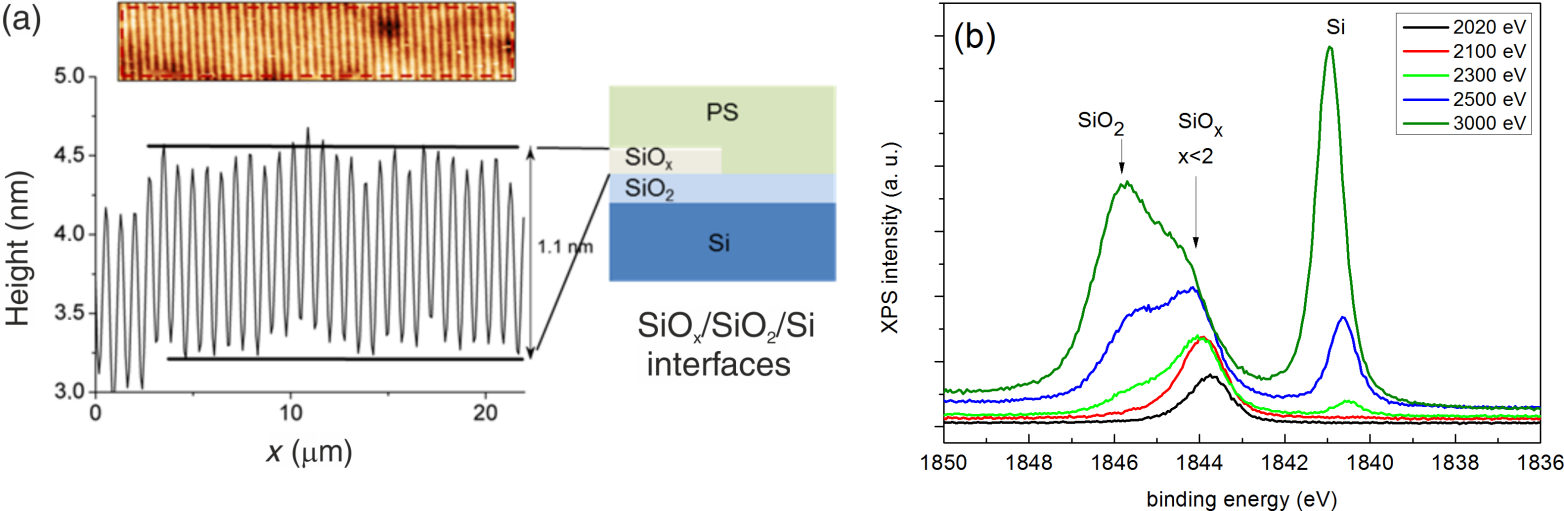
(a) AFM topography image and profile of the PS–OH brush annealed and cooled down in nitrogen after parallel oxidation nanolithography. A scheme is included showing the different regions. (b) HAXPES spectra of the Si 1s region at different photon energies.

Now, we focus the analysis on the sample prepared by PON. Figure 7a shows an AFM image of a chemical guiding pattern created by PON. The effect of the brush modification is an effective replication of the DVD pattern with modified regions (brighter in the AFM topography) slightly elevated (1.1 nm). The pitch of the DVD pattern is too large to achieve an aligned block/copolymer pattern. We used a DVD stamp in order to get a large area chemical guiding pattern useful for the HAXPES characterization. We also succeed in creating chemical guiding patterns with stamps of smaller pitch (see Supporting Information File 1, Figure S2), but in this case, we observed that the areas between lines were also chemically modified, preventing a block co-polymer chemical alignment. In any case, it was demonstrated in [11] that chemical patterns performed by local oxidation are very effective to align the block co-polymers if their geometrical dimensions are properly defined. Figure 7b shows the Si 1s spectra taken at different photon energies in the 2020–3000 eV range. At 2020 eV (black continuous line) only one feature is observed at about 1844 eV binding energy. At higher photon energies, two more lines are identified at about 1841 and 1846 eV binding energies, respectively, that become increasingly dominant for increasing photon energies. The 1841 and 1846 eV features correspond to the buried Si/SiO_2_ interface. Previous photoemission measurements performed at lower energy resolution using the Si 2p line conclude that most of the oxide grown using PON is purely stoichiometric, although contribution from Si lower oxidation states may be present [24]. In references [18,24–25] the Si 2p spin–orbit splitting is not resolved (compare to Figure S3 in Supporting Information File 1), which can preclude the observation of additional features in the region corresponding to the oxide.

In our case, we have selected the Si 1s line instead because of the higher interfacial sensitivity with photons above 2020 eV as compared to the Si 2p line. At 2020 eV, the corresponding kinetic energies are about 180 eV and 1920 eV for Si 1s and Si 2p, respectively, and the corresponding inelastic mean free path for electrons are 0.7 nm (Si) and 0.9 nm (SiO_2_) at 180 eV and 4.1 nm (Si) and 4.9 nm (SiO_2_) at 1920 eV, respectively [26]. Thus, we can continuously follow the emergence of the three features as a function of the increasing probing depth from the surface with the Si 1s lines. In the case of the Si 2p line, photon energies above 200 eV should be used, which could not be achieved at the KMC-1 beamline.

We can thus conclude that the 1844 eV line emerges from a region located on top of the Si/SiO_2_ interface, as schematized in Figure 7a, since it is the first feature that appears at the lower photon energy used. In addition, since the feature at 1844 eV exhibits a lower binding energy as compared to the 1846 eV counterpart, it can be assigned to a substoichiometric oxide layer (SiO*_x_*), with partially oxidized silicon. Both lines are shifted due to charging induced by the grown oxide layers [25]. No relevant changes are observed in the C 1s spectra taken at the same photon energies (apart from charging), although they do not correspond to the same probing depths, since the associated kinetic energies are above 1735 eV for C 1s eV, with inelastic mean free paths above 3.8 nm (see Supporting Information File 1, Figure S4).

It is worth mentioning that the use of brush layers of different thickness would produce a different phenomenology. For example, it is known that the use of brush layers thicker than 5 nm inhibits the interaction of the block copolymer with the substrate [27] and that for thinner layers, the surface neutralization provided by the brush layers is highly dependent on the polarity of the underlying substrate [28].

## Conclusion

We have demonstrated that HAXPES using synchrotron radiation is a powerful spectroscopic tool to explore the chemical properties of surfaces and buried interfaces of brush layers for directed self-assembly of block copolymers, since valuable chemical information as a function of probing depth can be obtained.

The choice of the correct cooling of annealed PS–OH brush layers is of paramount importance in order to obtain an optimal DSA process. HAXPES characterization shows an increase in intensity in the energy range corresponding to hydroxyl bonds when the brush is cooled down in the presence of oxygen. This is consistent with the change in the chemical affinity of the brush layer with the BCP experimentally observed in DSA.

With regard to the functionalization of the PS–OH brush layers, it has been proven that oxygen plasma exposure activates the brush layers by generating diverse carbon–oxygen bonding which promotes higher affinity to PMMA blocks. Electron beam exposure increases sp^2^ bonding, promoting higher affinity to PS blocks that might be explained by cross-linking of PS. In the case of parallel oxidation nanolithography, HAXPES provides experimental evidence of the existence of a substoichiometric oxide between the brush layer and the SiO_2_/Si substrate.

## Experimental

### Preparation and chemical modification of brush layers

The starting substrates were <100> silicon wafers (p-type silicon of 4–40 Ω·cm resistivity) with a native silicon oxide layer on top. A thin film of hydroxyl-terminated polystyrene (PS–OH, *M*_n_ = 4.5 kg/mol and PDI = 1.09, purchased from Polymer Source), was deposited by spin-coating and annealed on the silicon substrate. Two different annealing conditions were used: (i) 260 °C in nitrogen atmosphere for 5 min and cooling in air and (ii) 230 °C in nitrogen atmosphere for 5 min and cooling in nitrogen.

The first DSA process is based on using EBL with a subsequent substrate functionalization with oxygen plasma exposure in order to chemically modify the brush layer and thus make it slightly affine to the other BCP domain (see top of Figure 1). In a first step, the brush is grafted on top of the activated silicon substrate and it is annealed. Then, the non-grafted brush layer is rinsed away by dipping the sample into toluene for 5 min in an ultrasound bath. Consequently, the EBL is performed on an 80 nm thick PMMA resist and after the development, the sample is briefly exposed to oxygen plasma in order to chemically modify the exposed areas and thus change their chemical affinity.

The two other processes are based on direct writing methods, thus avoiding the use of a resist. In the first case, the sample was exposed directly to an electron beam which modifies the chemical affinity of the PS–OH brush layer (see middle of Figure 1) [12]. The third method (PON) [18], shown in the bottom of Figure 1, is performed by contacting a conductive mold with the PS–OH surface while applying a voltage under conditions of high humidity. The stamp consists of a 1 cm^2^ piece of a DVD replica made with PDMS and coated with 100 nm gold film evaporated in high vacuum. The stamp surface presents parallel hillocks 320 nm wide and spaced 740 nm. The height of the protrusions is 40 nm. To transfer the patterns from the stamp to the substrate a 35–40 V bias voltage (sample positive) for a time ranging between 40 and 180 s was applied while the stamp was gently (50 kPa) pressed upon the substrate. Relative humidity was kept above 70%. These parameters are similar to the parameters used to perform an oxidation scanning probe lithography (SPL) experiment [29]. The authors have already demonstrated the efficiency of oxidation SPL to create chemical guiding patterns for DSA [11].

After defining the chemical guiding patterns, lamellar poly(styrene-*b*-methyl methacrylate) (PS-*b*-PMMA) dissolved in a 1.15% (w/w) toluene solution was spin-coated onto the substrates and annealed at 200 °C for 20 min in air. The obtained films exhibit a thickness of about 36 nm with the used conditions (2750 rpm for 60 s). In addition, and in order to characterize the interface between the oxygen modified and unmodified substrates with PMMA domains, ≈20 nm thick PMMA (*M*_n_ = 30 kg/mol) layers were deposited by spin-coating on top of them.

### Characterization techniques

The HAXPES experiments were performed at the HIKE end-station located at the KMC-1 beamline at the BESSY II synchrotron of the Helmholtz Zentrum Berlin für Materialien und Energie in Berlin (Germany) [30–31]. Monochromatic radiation in the 2020–6000 eV photon energy range was used in our experiments, impinging the sample surface at grazing incidence. Photo-emitted electrons were collected with a SCIENTA R4000 high-resolution hemispherical analyzer at near normal emission, with an upper limit in kinetic energy of 10,000 eV. Experiments were performed in an ultrahigh vacuum chamber with a base pressure in the high 10^−9^ mbar range. To prevent beam damage, measurements were taken at different locations on the sample. In addition, the radiation was stopped (beam shutter closed) when spectra were not acquired (e.g. in case of monochromator setting change, change of sample position, etc.). Ex situ XPS experiments were performed at room temperature with a SPECS PHOIBOS 150 hemispherical analyzer using monochromatic Al Kα (1486.6 eV) radiation as an excitation source at a base pressure in the 10^−9^ mbar range.

As compared to previously reported XPS experiments performed with conventional sources [32], it is important to note that: (i) our results have been obtained with high energy resolution (see Supporting Information File 1, Figure S1), (ii) no external charge neutralization source has been used to compensate for charging effects due to the insulating character of the polymers and (iii) the binding energies have been referenced to the Si 2p_3/2_ line of the underlying pure silicon substrate, with a binding energy of 99.3 eV [33], as independently determined by XPS and corresponding to a moderately p-doped sample as well as to the Si 2p_3/2_ line of a clean silicon reference sample [34]. Shifts induced by recoil effects [35–36] and band bending [37] are not considered here since we are interested in the relative position of the photoemission lines (rigid shits) rather than on their absolute binding energies.

The SEM images shown in this work have been obtained with an AURIGA system from Zeiss and a Dimension Icon atomic force microscopy (AFM) from Bruker. The film thickness was determined by means of X-ray reflectometry (XRR) performed using a Philips X’Pert Pro MRD diffractometer equipped with a parabolic mirror using Cu Kα radiation (1.54187 Å).

## Supporting Information

Simulated proportion of the O–C=O contribution to the normalized C 1s spectrum as a function of the photoelectron kinetic energy. AFM images of a mold oxidation stamp fabricated on glass by NIL and metalized (5 nm Cr/70 nm Au) and corresponding parallel oxidation pattern on PS–OH. Photoemission spectra of the Si 2p line taken with 2020 eV photons of a PS/SiO_2_/Si sample. Photoemission spectra of the C 1s lines of the PON sample taken with 2020 and 3000 eV photons.

File 1 Additional experimental results.
