# Supplementary material for: Identifying the nature of surface chemical modification for directed self-assembly of block copolymers
Source: Beilstein J Nanotechnol. 2017 Sep 21;8:1972–81. doi: 10.3762/bjnano.8.198 (PMC5629386; doi:10.3762/bjnano.8.198)
Supplement: File 1 — Additional experimental results. [file Beilstein_J_Nanotechnol-08-1972-s001.pdf]

Supporting Information

for

Identifying the nature of surface chemical modification for directed self-assembly of block copolymers

Laura Evangelio[1,2], Federico Gramazio[2], Matteo Lorenzoni[1], Michaela Gorgoi[3], Francisco Miguel Espinosa*[4], Ricardo García[4], Francesc Pérez-Murano[*1] and Jordi Fraxedas[*2]

Address: [1]Institut de Microelectrònica de Barcelona (IMB-CNM, CSIC), C/Til·lers, Campus Universitat Autònoma de Barcelona, Cerdanyola del Vallès, Barcelona, Spain, [2]Catalan Institute of Nanoscience and Nanotechnology (ICN2), CSIC and BIST, Campus UAB, Bellaterra, 08193, Barcelona, Spain, [3]Institute for Nanospectroscopy, Energy Materials In-situ Laboratory (EMIL), Helmholtz Zentrum Berlin für Materialien und Energie GmbH, Albert Einsteinstrasse 15, 12489 Berlin, Germany and [4]Instituto de Ciencia de Materiales de Madrid (ICMM-CSIC), Sor Juana Inés de la Cruz, 3, Cantoblanco, 28049 Madrid, Spain

Email: Francesc Pérez-Murano - francesc.perez@csic.es; Jordi Fraxedas - jordi.fraxedas@icn2.cat

* Corresponding author

Additional experimental results

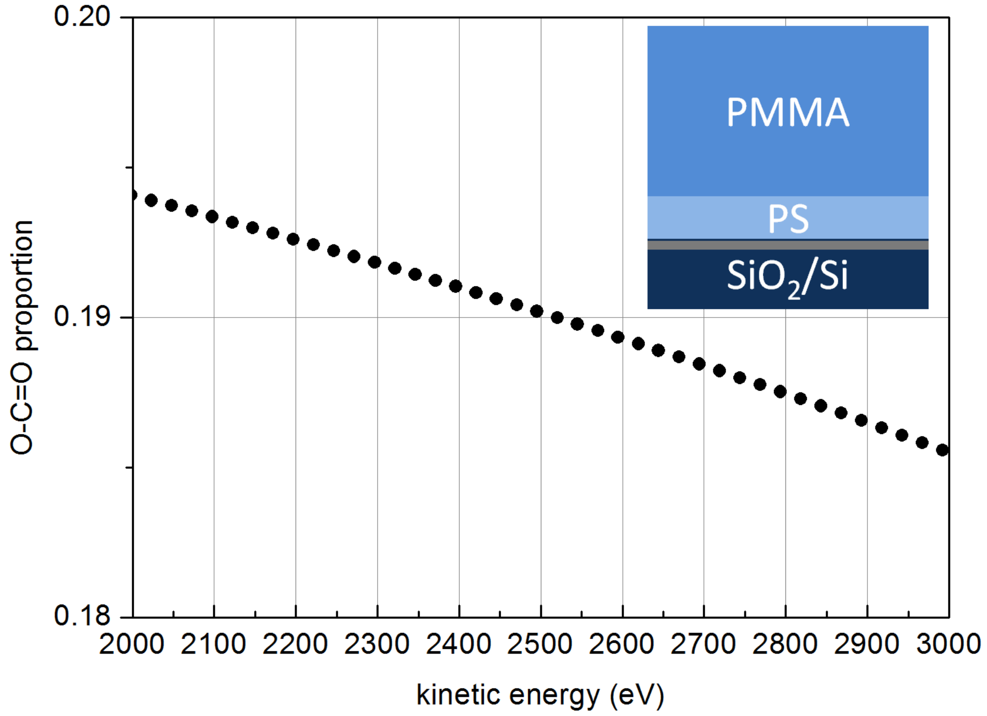

Figure S1: Simulated proportion of the O-C=O contribution to the C1s spectrum (normalized to 1) as a function of the photoelectron kinetic energy.

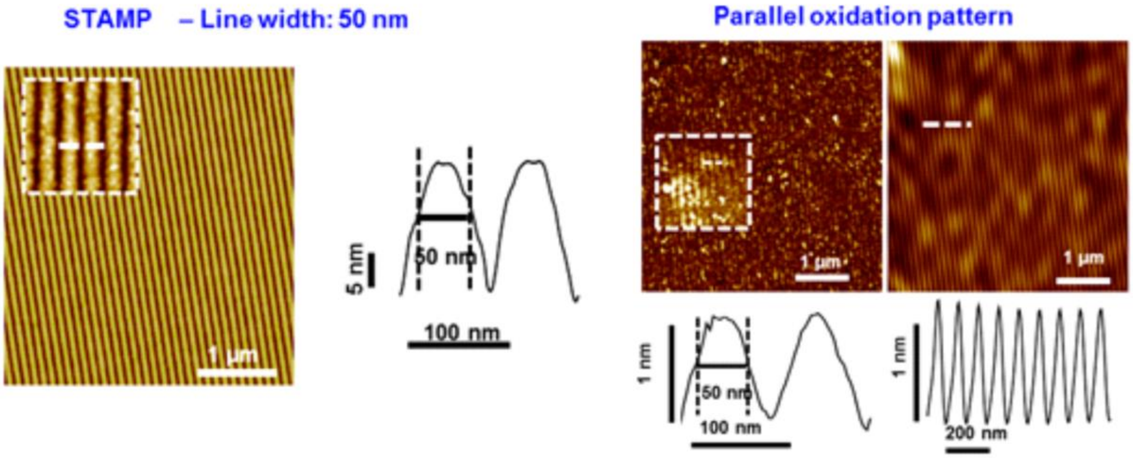

Figure S2: AFM images of a mold oxidation stamp (left panel) and corresponding parallel oxidation pattern on PS-OH (right panel). The stamp was fabricated on glass by nanoimprint lithography (NIL) and later metalized (5 nm Cr-70 nm Au). Parallel oxidation parameters: V = 56 V (sample positive), I = 0.02 A, time = 1 min 30 s.

The inset to **Figure S2** shows a scheme of a layer of PMMA with a thickness d_{PMMA} covering homogeneously an underlying PS layer with a thickness d_{PS} on top of a silicon substrate. In normal emission the photoemission intensity of the lines (e.g., C1s) will follow the well-known expressions [S1]:

$$I_{PMMA} \propto \mu_{PMMA} \left[1 - e^{-d_{PMMA}/\mu_{PMMA}}\right]$$

$$I_{PS} \propto \mu_{PS}\, e^{-d_{PMMA}/\mu_{PMMA}} \left[1 - e^{-d_{PS}/\mu_{PS}}\right]$$

where μ_{PMMA} amd μ_{PS} stand for the inelastic mean free path in PMMA and PS, respectively, and I_{PMMA} and I_{PS} correspond to the intensities of the peaks related to PMMA and PS, respectively (see main text for details). The proportion *P* of the O-C=O contribution to the spectrum can be calculated from the expression [S2]:

$$P = \frac{I_{PMMA}}{5I_{PMMA} + 8I_{PS}}$$

For large d_{PMMA} values and low d_{PS} values the PS contribution is negligible and the nominal 0.2 value is obtained. The proportion *P* is a function of the kinetic energy since the inelastic mean free paths vary with kinetic energy. For the calculation in **Figure S2** we have used d_{PMMA} = 20 nm and d_{PS} = 5 nm and the functional dependence of the inelastic mean free path has been obtained from ref S3. The proportion decreases for higher values of the kinetic energy as a result of the increasing probing depth (higher mean free path values). The calculated variation in the kinetic energy range of interest is lower than the observed values obtained in the experiments, which calls for a larger contribution of the underlying PS layer through inhomogeneous coverage (dewetting).

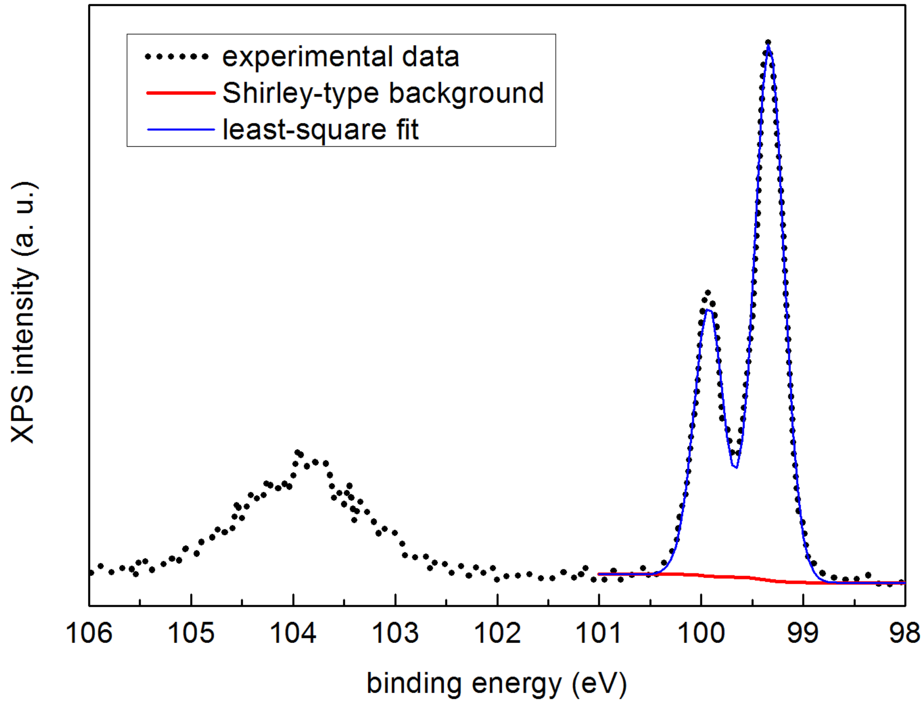

Figure S3: Photoemission spectra of the Si2p line taken with 2020 eV photons (lowest photon energy available) of a PS/SiO_2/Si sample. Both the signals arising from bulk Si (at 99.3 eV binding energy) and from SiO_2 (at about 104 eV binding energy) are displayed. A least-square fit of the Si2p line after a Shirley-type background subtraction gives a spin-orbit splitting of 0.59 eV, a branching ratio of 2:1 (2p3/2:2p1/2) and a FWHM of 0.34 eV, highlighting the actual high resolution at use. Identical results are obtained for SiO2/Si substrates, indicating the negligible effect of the PS brush on scattering of photoelectrons.

A silicon stamp consisting of lines with 200 nm and 300 nm pitch was fabricated by electron beam lithography and wet chemical etching, following the process described in [S4], that result in pyramid-like lines. This silicon stamp was replicated in PDMS. The PDMS replica was metallized by evaporation of chromium and gold. The resulting triangular features protruded 22 nm. The widest area patterned reached 0.2 mm x 0.2 mm. These stamps were useful to create patterns on the brush layers (See **Figure S3**) but they were not able to align the block copolymer after self-assembly because the height of the lines was too low, producing a chemical modification of the whole patterned area.

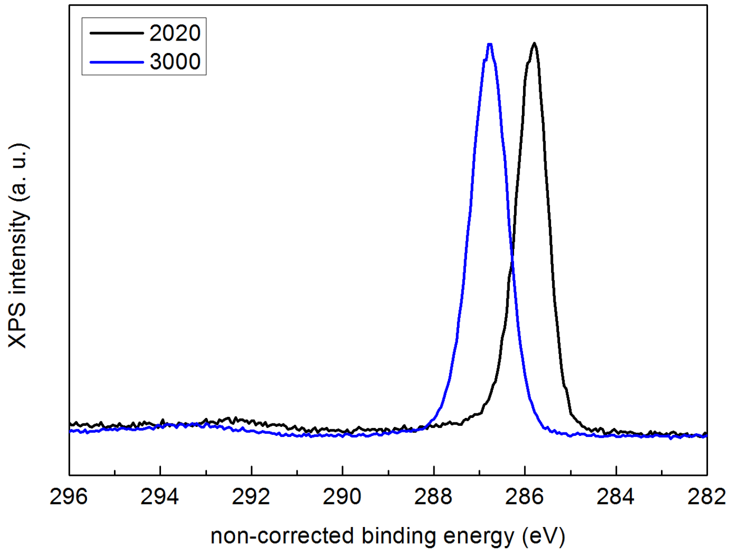

Figure S4: Photoemission spectra of the C1s lines of the PON sample taken with 2020 eV (continuous black line) and 3000 eV (continuous blue line) photons, respectively. The spectra have been normalized to their maxima.

References

[S1] Himpsel, F. J.; McFeely, F. R.; Taleb-Ibrahimi, A.; Yarmoff, J. A; Hollinger, G. *Phys. Rev. B* **1988**, *38* (9), 6084-6096.

[S2] Ton-That, C.; Shard, A. G.; Teare, D. O. H.; Bradley, R. H. *Polymer* **2001**, *42*, 1121–1129.

[S3] Tanuma, S.; Powell, C. J.; Penn, D. R. *Surf. Interface Anal*. **1991**, *17* (13), 911–926.

[S4] Fernandez-Cuesta, I.; Borrisé, X.; Retolaza, A.; Merino, S.; Mendels, D. A.; Hansen, O.; Kristensen, A.; Pérez-Murano, F. *Microelectronic Engineering* **2008**, 85(5), 838-841.
